# Liverpool Epidemic Strain Isolates of Pseudomonas aeruginosa Display High Levels of Antimicrobial Resistance during Both Planktonic and Biofilm Growth

**DOI:** 10.1128/spectrum.01024-22

**Published:** 2022-06-06

**Authors:** Mara C. Goodyear, Nicole E. Garnier, Roger C. Levesque, Cezar M. Khursigara

**Affiliations:** a Department of Molecular and Cellular Biology, University of Guelph, Guelph, Ontario, Canada; b Institut de Biologie Integrative et des Systems (IBIS), Département de Microbiologie-Infectiologie et d'Immunologie, Université Laval, Laval, Quebec, Canada; INTHERES

**Keywords:** *Pseudomonas aeruginosa*, Liverpool epidemic strain, biofilms, antibiotic resistance

## Abstract

Eight isolates of the Liverpool epidemic strain (LES) of Pseudomonas aeruginosa have previously been characterized using comparative genomics and preliminary phenotypic assays. Here, we extend the characterization of these clinically relevant P. aeruginosa isolates with planktonic and biofilm growth assays and analysis of antibiotic susceptibility for both planktonic and biofilm cultures. Laboratory strains PAO1 and PA14 were included as comparator strains. Antibiotic susceptibility to eight classes of antibiotics was determined. MICs were determined to measure susceptibility of planktonic cultures, and minimum biofilm eradication concentration (MBEC) assays were used to estimate levels of resistance during the production of biofilm. LES isolates had high levels of resistance compared with laboratory reference strains when grown planktonically (up to nine 2-fold dilutions higher), and resistance was increased in the biofilm mode of growth. Measurements of biofilm biomass in the MBEC assays showed that certain isolates often show increased biofilm biomass in the presence of antibiotics.

**IMPORTANCE** Pseudomonas aeruginosa is an opportunistic pathogen with high intrinsic antibiotic resistance. This resistance is typically increased in clinical isolates through adaptations to the host and production of small-colony variants (SCVs) and when P. aeruginosa forms biofilms, which are surface-attached communities that are protected by a self-produced matrix. Understanding the combination of SCVs, biofilm production, and the diversity of drug resistance phenotypes in clinical isolates can lead to improved treatments for P. aeruginosa infections.

## INTRODUCTION

Pseudomonas aeruginosa is a highly antibiotic-resistant pathogen, often displaying resistance to multiple types of antibiotics (1, 2). P. aeruginosa causes chronic lung infections in people with cystic fibrosis (pwCF), and these individuals often receive multiple rounds of antibiotic treatments (3, 4). Antibiotics from multiple classes are used individually and in combination in the treatment of P. aeruginosa lung infections, including those that target bacterial cell wall synthesis, protein synthesis, DNA replication, and outer membrane stability. P. aeruginosa possesses various mechanisms of resistance that allow it to survive treatment with many of these antibiotics. These resistance mechanisms include antibiotic-inactivating enzymes, antibiotic efflux pumps, and the ability of P. aeruginosa to form biofilms, which can further enhance antibiotic resistance (1, 2, 5).

Biofilms are communities of bacteria adhered to a surface and surrounded by a matrix of self-produced extracellular polymeric substances (EPS), which include polysaccharides, proteins, and DNA (6, 7). The biofilm matrix acts as a barrier and can prevent antibiotics from reaching the cells within the biofilm (8–11). Nutrient and oxygen gradients within biofilms can lead to metabolically inactive cells within biofilms, which are less susceptible to antibiotics that target active processes such as cell wall and protein synthesis (12–14). Small-colony variants (SCVs) of P. aeruginosa have also been isolated from biofilms and can display decreased antibiotic susceptibility (15, 16). Due to these factors, biofilms are often more resistant to antibiotics than cells growing planktonically (8). Susceptibility testing of bacteria is often done by determining the MIC using planktonic cultures. Other assays, such as the minimum biofilm eradication concentration (MBEC) assay, determine the lowest concentration of antibiotic able to eliminate a preestablished biofilm and represent excellent methods for measuring differences in resistance between planktonic and biofilm bacteria (17).

Clinical isolates of P. aeruginosa can display higher levels of resistance than commonly studied laboratory strains, and some groups of clinical isolates show further increased resistance (2, 18). For example, isolates of the Liverpool epidemic strain (LES) of P. aeruginosa have been shown to have higher levels of antibiotic resistance than nonepidemic P. aeruginosa cystic fibrosis (CF) isolates (19). The LES was the first epidemic strain of P. aeruginosa to be described and was discovered because isolates from multiple individuals in a CF clinic setting were displaying high levels of resistance to the cephalosporin ceftazidime (20). While MICs for a limited number of antibiotics have been reported for various LES isolates, different methods of determining the MIC values have been used (21–25), and the levels of resistance in LES isolates growing as biofilms have not been investigated in detail.

The goal of this study was to characterize the planktonic and biofilm growth of LES isolates and laboratory strains and compare their resistance in both modes of growth. We used high-throughput 96-well plate-based assays to characterize eight LES isolates and the laboratory strains PAO1 and PA14. Growth curves, biofilm assays, MIC assays, and MBEC assays were completed to produce growth and susceptibility profiles. The LES isolates investigated have previously been studied using comparative genomics and some phenotypic assays (21, 26–28). Our analyses show that overall, LES isolates have higher levels of resistance than PAO1 when tested with a panel of 14 antibiotics using the broth microdilution MIC method. A subset of LES isolates had increased resistance to β-lactam antibiotics, while for some other classes of antibiotics, all LES isolates had higher MIC values. MBEC assays revealed that resistance was further increased in the biofilm mode of growth and allowed us to determine trends in biofilm biomass changes upon exposure to antibiotics. These results provide a baseline characterization of these isolates and lead to questions about the mechanisms responsible for these different levels of resistance.

## RESULTS

### Planktonic growth and biofilm formation of LES isolates.

**FIG 1:**
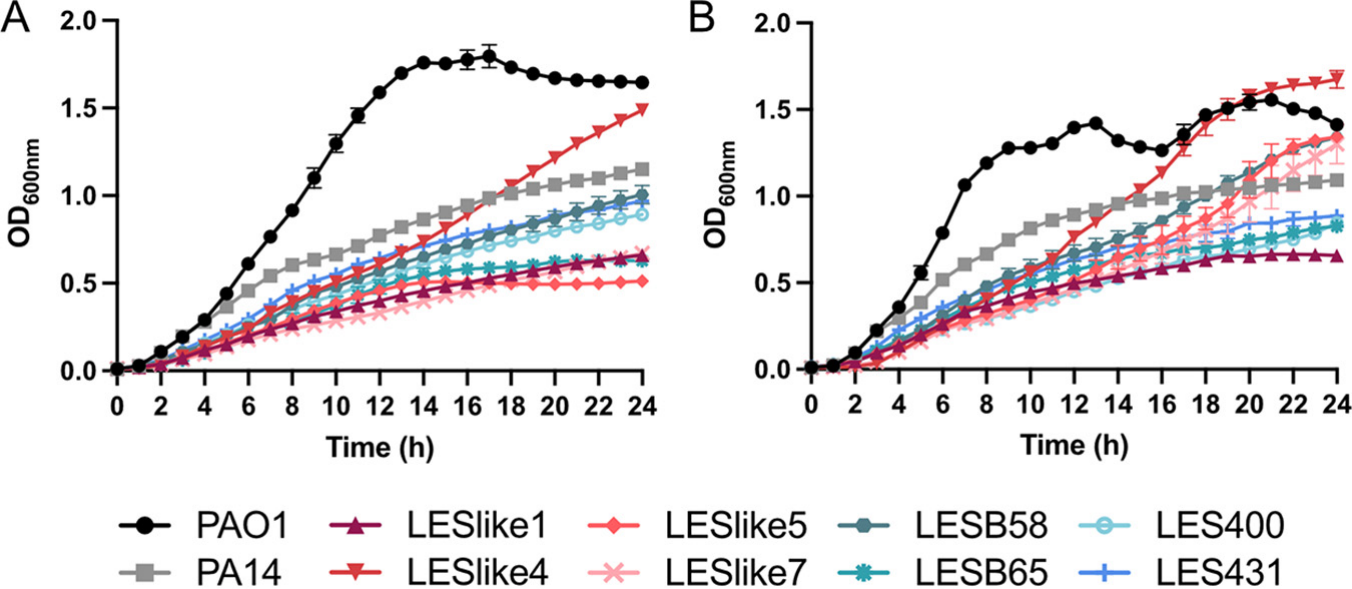
Planktonic growth curves. Shown are growth curves over 24 h of PAO1, PA14, and LES isolates in (A) TSB and (B) CAMHB. OD_600_ values are reported as the mean ± standard deviation (SD) from three biological replicates (eight technical replicates each).

To establish baseline information about the growth of LES isolates, planktonic growth curves and biofilm assays were completed. Assays were completed in Trypticase soy broth (TSB), which is commonly used for culturing Pseudomonas and cation-adjusted Mueller-Hinton broth (CAMHB), which is recommended for use in susceptibility testing (29). Overall, strains/isolates showed similar growth in both media (Fig. 1). Only LESlike5 and LESlike7 showed major differences, with optical density at 600 nm (OD_600_) values approximately two times higher in CAMHB after 24 h compared to those in TSB. In both TSB and CAMHB, PAO1 reached higher OD_600_ values faster than the LES isolates and had higher final OD_600_ values than most isolates after 24 h, especially in TSB. For example, in TSB, PAO1 reached an OD_600_ of 1.0 by 9 h, while some LES isolates reached an OD_600_ close to 1.0 by 24 h (LESB58, LES400, and LES431), and other LES isolates never reached an OD_600_ of 1.0 (LESlike1, LESlike5, LESlike7, and LESB65).

**FIG 2:**
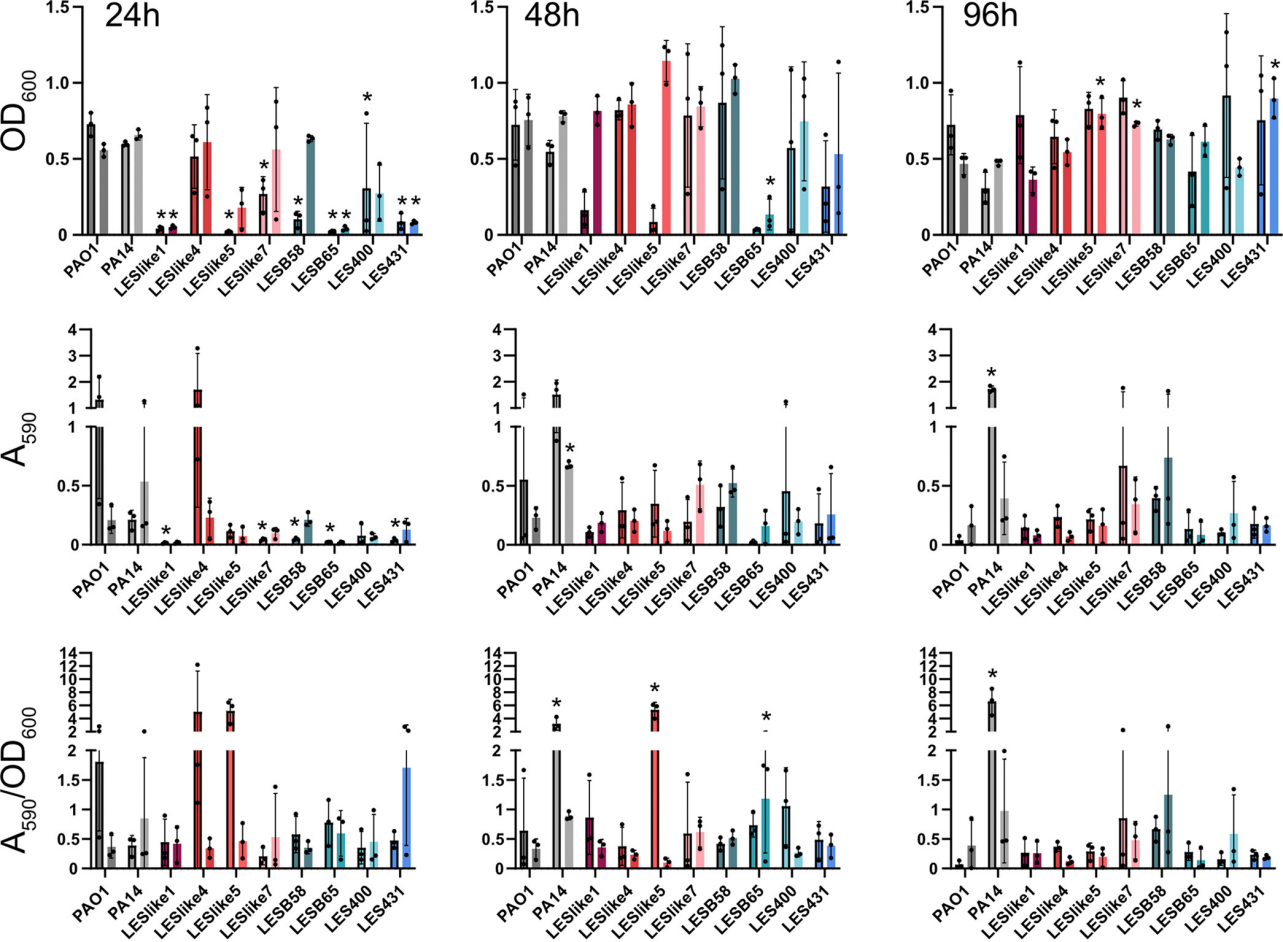
Biofilm assays for LES isolates and laboratory strains. Biofilms were cultured for 24, 48, and 96 h. At each time point, the planktonic growth (OD_600_) and biofilm biomass (*A*_590_) were measured. Biofilm biomass was then normalized to planktonic growth (*A*_590_/OD_600_). Assays were completed in both TSB and CAMHB, and results for both media are shown on each graph. For each strain/isolate, the left bar is for TSB (bar with black outline), and the bar for CAMHB is on the right. Values are reported as the mean ± SD from three biological replicates (eight technical replicates each). *, *P* < 0.05, one-way ANOVA with Dunnett’s multiple-comparison test. Each strain/isolate was compared to PAO1 at the same time point and in the same medium.

Biofilm assays were completed at three time points (24, 48, and 96 h) to uncover any temporal changes in biofilm formation. Biofilm formation was determined by staining with crystal violet (CV), which detects all biofilm biomass (e.g., cells and matrix components). At each time point, the biofilm biomass and planktonic growth were measured, and biofilm biomass was normalized to planktonic growth to account for any differences in growth rates under the conditions of the biofilm assays. Planktonic growth was variable after 24 h in both media, and LES isolates showed overall lower OD_600_ values than PAO1, similar to what we observed in the growth curves (Fig. 2, top row). By 96 h, planktonic growth values were more similar across the isolates and closer to those of PAO1 (Fig. 2, top row). In TSB, PAO1 biofilm biomass decreased across the time points, while PA14 biomass increased (Fig. 2, middle row). Overall, LES isolates had low levels of biomass across all three time points (Fig. 2, middle row). When normalized to planktonic growth, the main change observed was an increase in biofilm formation in LESlike5 at 48 h (Fig. 2, bottom row). In CAMHB, when biofilm formation was normalized to planktonic growth, LESB65 had significantly higher biofilm formation at 48 h than PAO1. In CAMHB, the increases in biofilm when normalized to planktonic growth were not observed for PA14 and LESlike5, as observed in TSB.

### LES isolates display increased resistance to multiple classes of antibiotics.

**FIG 3:**
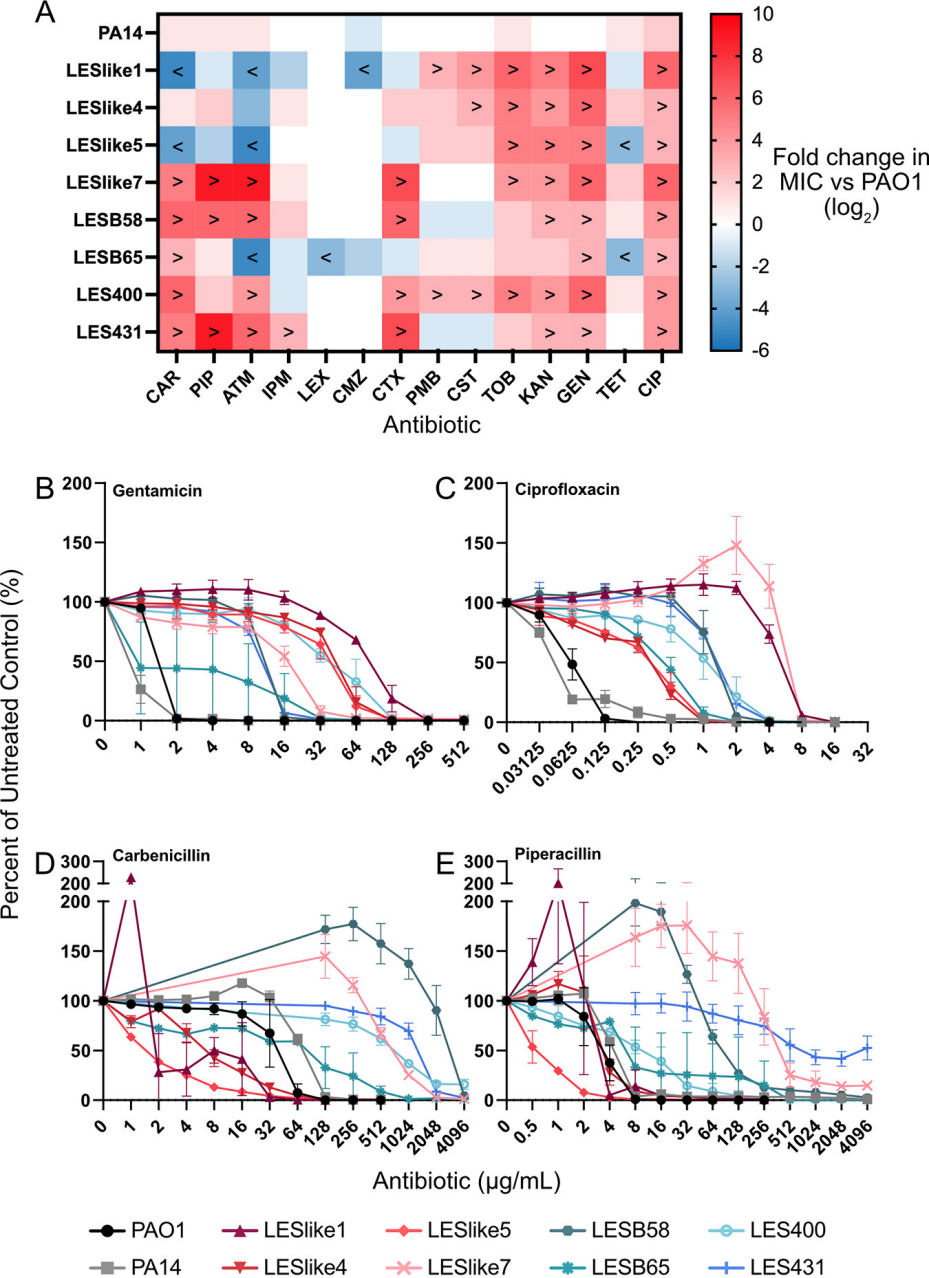
Comparison of LES and PAO1 MICs. (A) The heat plot shows the log_2_ fold change between the MICs for PA14 and LES isolates and those for PAO1. Red squares indicate strain-antibiotic combinations with a MIC greater than that of PAO1, and blue indicates strain-antibiotic combinations with a MIC lower than that of PAO1. White squares indicate strains/isolates that had the same MIC as PAO1. The < and > symbols indicate where the LES isolates had MIC values that were three or more 2-fold dilutions lower (<) or higher (>) than the PAO1 MIC. (B to E) Percentage of survival graphs representative of the trends in MIC values observed in the comparison of LES and PAO1 MICs. All LES isolates had higher MIC values for aminoglycoside antibiotics such as gentamicin (B) and for ciprofloxacin (C) than PAO1. LESlike7, LESB58, LES400, and LES431 showed increased MIC values for β-lactam antibiotics such as carbenicillin (D) and piperacillin (E).

To determine the levels of antibiotic resistance in the LES isolates, MIC assays were performed. Bacteria were challenged with 14 antibiotics from eight different classes (Table 1). The selected antibiotics target different cellular structures and processes, including cell wall synthesis, the outer membrane, protein synthesis, and DNA replication. Some of the antibiotics tested are given to pwCF to treat P. aeruginosa infections. MICs determined for PAO1, PA14, and the LES isolates are listed in Table 2. PAO1 and PA14 had similar MICs within one 2-fold dilution of each other for all antibiotics except ciprofloxacin (two 2-fold dilutions apart). However, the LES isolates showed overall greater MIC values than PAO1, with MICs up to nine 2-fold dilutions higher. To visualize the differences between the MICs of PAO1 and the other strains tested, we created a heat plot, which shows the log_2_ fold change in MIC for each strain-antibiotic combination compared with PAO1 (Fig. 3A). The number of red squares in the heat plot readily shows the overall increased levels of resistance in the LES isolates. The values in the heat plot also indicate the number of 2-fold dilutions between the MIC value in PAO1 and the corresponding LES MIC. For example, a value of 6 for LESB58 carbenicillin (CAR) in the heat plot indicates that the MIC of LESB58 is six 2-fold dilutions higher than in PAO1 (>4,096 μg/mL versus 64 μg/mL). MIC values in the LES that are three or more 2-fold dilutions above or below the MIC of PAO1 are indicated on the heat plot with greater than and less than symbols (> or <). These changes in MIC values are of more interest than changes of only one or two 2-fold dilutions, which can be caused by variability in the assay and may not indicate a significant difference in resistance.

**TABLE 1:** Antibiotics used in susceptibility testing^*a*^

Class	Target(s)	Antibiotic (reference)	Use in CF treatment^*b*^
Penicillins	Cell wall synthesis	Carbenicillin (29)	NA
		Piperacillin (29)	Intravenous

Cephalosporins	Cell wall synthesis	Cefotaxime (29)	NA
		Cefmetazole (45)	NA
		Cephalexin (45)	Oral

Monobactams	Cell wall synthesis	Aztreonam (29)	Intravenous, inhaled

Penems	Cell wall synthesis	Imipenem (29)	Intravenous

Lipopeptides	Outer membranes	Polymyxin B (29)	NA
		Colistin (polymyxin E) (29)	Intravenous, inhaled

Aminoglycosides	30S ribosomal subunit, protein synthesis	Gentamicin (46)	Intravenous, inhaled
		Kanamycin (46)	NA
		Tobramycin (46)	Intravenous, inhaled

Tetracyclines	30S ribosomal subunit, protein synthesis	Tetracycline (29)	NA

Quinolones	DNA gyrase	Ciprofloxacin (29)	Oral, intravenous

a Classes and targets of antibiotics and the mode of administration for antibiotics used to treat P. aeruginosa in CF lung infections are listed.

b Described in more detail in reference 3. NA, not applicable.

**TABLE 2:** MIC values for PAO1, PA14, and LES isolates determined by the broth microdilution method

Strain/isolate	MIC (μg/mL) of^*a*^:													
	CAR	PIP	ATM	IPM	CMZ	CTX	LEX	GEN	KAN	TOB	PMB	CST	TET	CIP
PAO1	64	8	8	1	4,096	32	>4,096	2	128	0.5	1	1	32	0.125
PA14	128	16	16	1	2,048	32	>4,096	2	128	1	1	1	64	0.5
LESlike1	2	4	0.5	0.25	256	16	>4,096	256	4,096	32	8	16	16	8
LESlike4	128	32	1	2	>4,096	128	>4,096	128	2,048	16	4	8	128	1
LESlike5	4	2	0.25	1	4,096	16	>4,096	64	4,096	16	4	4	4	1
LESlike7	2,048	4,096	4,096	2	4,096	4,096	>4,096	128	2,048	8	1	1	128	8
LESB58	>4,096	512	512	4	>4,096	2,048	>4,096	16	1,024	2	0.5	0.5	64	2
LESB65	512	16	0.25	0.5	1,024	16	512	16	512	2	2	2	4	1
LES400	4,096	32	128	0.5	4,096	512	>4,096	128	2,048	16	8	8	64	2
LES431	2,048	>4,096	512	8	>4,096	>4,096	>4,096	16	1,024	2	0.5	0.5	32	2

a Shown are the median MIC values for *n* = 3 biological replicates (three technical replicates each). CAR, carbenicillin; PIP, piperacillin; ATM, aztreonam; IPM, imipenem; CMZ, cefmetazole; CTX, cefotaxime; LEX, cephalexin; GEN, gentamicin; KAN, kanamycin; TOB, tobramycin; PMB, polymyxin B; CST, colistin; TET, tetracycline; CIP, ciprofloxacin.

The heat plot facilitated observation of patterns in resistance levels. All LES isolates had higher MICs than PAO1 when challenged with aminoglycosides (gentamicin [GEN], kanamycin [KAN], and tobramycin [TOB]) and the fluoroquinolone ciprofloxacin (Fig. 3B and C). For LES isolates that had decreased resistance, LESlike1, LESlike5, and LESB65 were the only isolates that had MIC values three or more 2-fold dilutions lower than PAO1 (for some β-lactams and tetracycline [TET]). The heat plot also shows a pattern of increased MICs for cell-wall-targeting β-lactam antibiotics (carbenicillin [CAR], piperacillin [PIP], aztreonam [ATM], and cefotaxime [CTX]) only in LES isolates LESlike7, LESB58, LES400, and LES431 (Fig. 3D and E). For these four antibiotics, these LES isolates had MIC values that were an average of six 2-fold dilutions higher than those in PAO1. LESlike7, LESB58, LES400, and LES431 all had MICs between four and nine 2-fold dilutions higher than those of PAO1 for these β-lactams (except LES400 and piperacillin [PIP]).

### LES biofilms have increased resistance to antibiotics.

**FIG 4:**
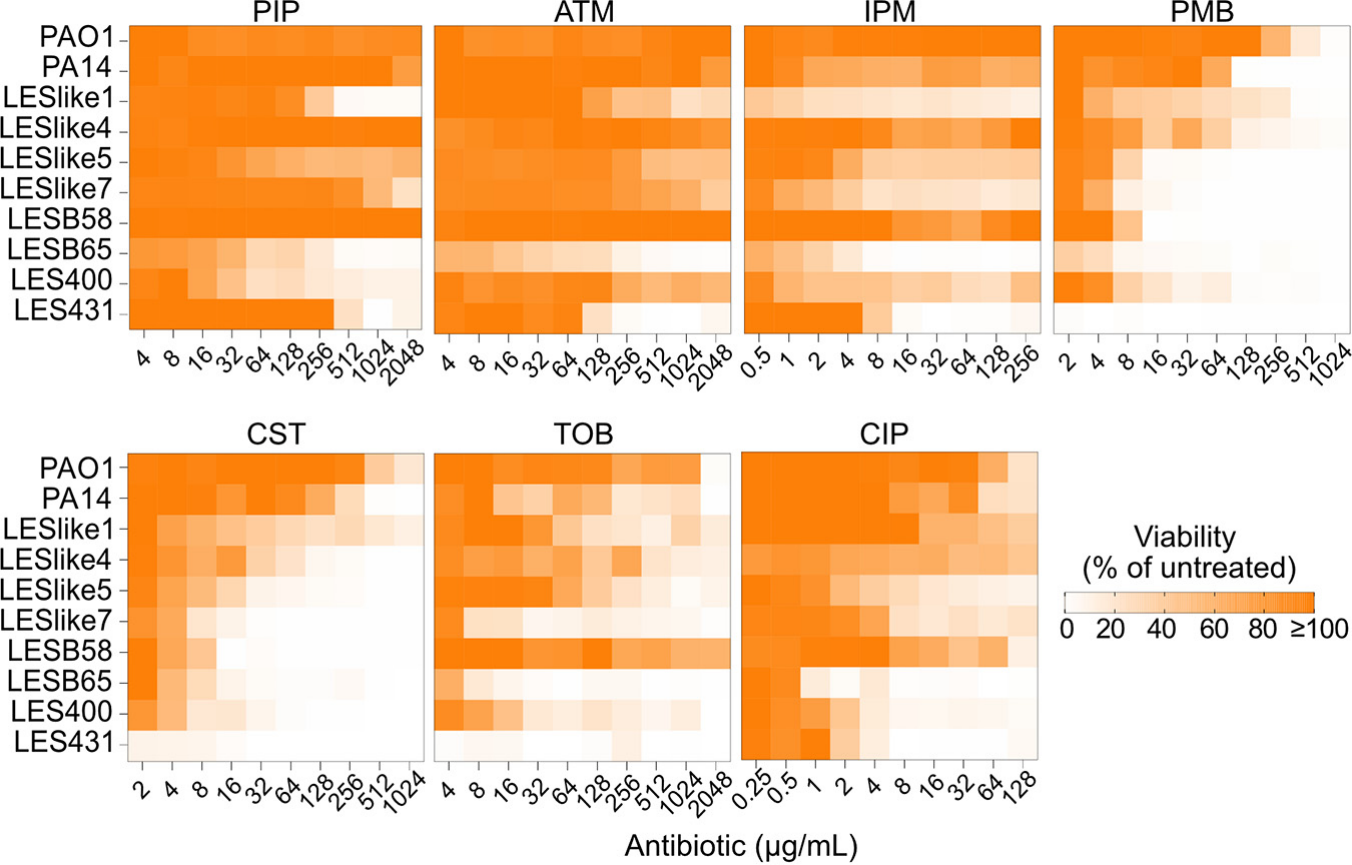
Biofilm viability after antibiotic challenge. Heat plots show the viability (percentage of untreated control) of biofilms across the concentration ranges tested in the MBEC assays as determined after the recovery phase.

**FIG 5:**
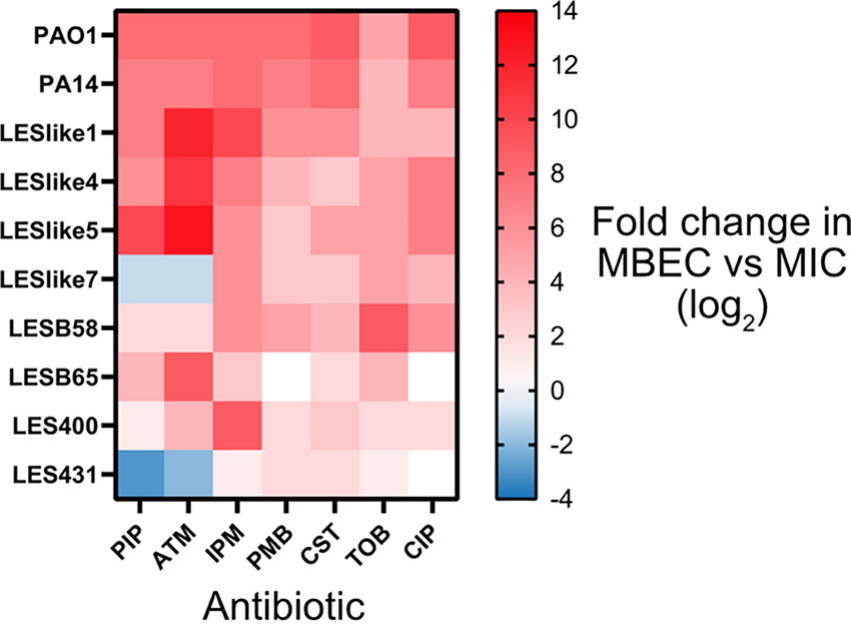
Comparison of biofilm (MBEC) and planktonic (MIC) susceptibility. The heat plot shows the log_2_ fold change between the MBEC and MIC for each strain-antibiotic combination. For strain-antibiotic combinations where there was a range in the MBEC values between the two biological replicates, the lowest MBEC value observed was used to compare with the MIC. Red squares indicate strain-antibiotic combinations where the MBEC was greater than the MIC (greater resistance in the biofilm mode of growth), and blue indicates strain-antibiotic combinations where the MBEC was lower than the MIC (greater resistance in the planktonic mode of growth). White squares indicate that the MIC and MBEC values were equivalent.

Seven antibiotics from six different classes were chosen to test in MBEC assays. These antibiotics included six that are used in the treatment of P. aeruginosa in lung infections (piperacillin [PIP], aztreonam [ATM], imipenem [IPM], colistin [CST], tobramycin [TOB], and ciprofloxacin [CIP]) and an additional polymyxin, polymyxin B (PMB). A difficulty in comparing multiple strains in the MBEC assay is whether the strains form equivalent amounts of biofilm in the growth phase before they are challenged with antibiotic. To compare the biofilms established in the growth phase, others have compared the number of colony-forming units per peg (CFU/peg) for the different strains (30). The CFU/peg values of the biofilms of PAO1, PA14, and the LES isolates were similar after 24 h of growth, and so the same inoculation method and growth time were used for all strains/isolates (see Fig. S1 in the supplemental material). The MBEC values determined are listed in Table 3. Many of the biofilms remained viable even at the highest concentrations tested (Fig. 4). To compare the resistance of planktonic and biofilm cultures, we made a heat plot to compare the MBEC values with the corresponding MIC value for each strain and antibiotic combination (Fig. 5). Overall, the MBEC values were higher than the MIC values, with MBEC values up to 13 2-fold dilutions higher than the MIC. This largest increase in MBEC value was observed for LESlike5 and aztreonam, where the MIC value is 0.25 μg/mL and the MBEC value is 2,048 μg/mL (~8,000 times higher). Of the 70 strain and antibiotic combinations tested in the MBEC assay, 31 resulted in MBEC values that were at least 50 times higher than the corresponding MIC values. For some isolates that were not resistant to β-lactams compared to PAO1 in the MIC assays, we observed higher MBEC values (LESlike1, LESlike4, and LESlike5).

**TABLE 3:** MBEC values for PAO1, PA14, and LES isolates

Strain/isolate	MBEC (μg/mL) of^*a*^:						
	PIP	ATM	IPM	PMB	CST	TOB	CIP
PAO1	>2,048	>2,048	>256	256 to 512	512 to 1,024	16 to 2,048	64 to >128
PA14	>2,048	>2,048	>256	128	256	16 to 2,048	64 to >128
LESlike1	512 to >2,048	≥2,048	>256	512	>1,024	512 to 2,048	>128
LESlike4	>2,048	>2,048	>256	64 to 1,024	64 to 128	512 to >2,048	>128
LESlike5	>2,048	>2,048	64 to >256	32 to 64	128 to 512	512 to >2,048	>128
LESlike7	>2,048	>2,048	128 to >256	8 to 32	8 to 32	256 to 2,048	>128
LESB58	>2,048	>2,048	>256	16	8 to 16	1,024 to 2,048	>128
LESB65	256 to >2,048	128 to 1,024	4 to 16	2 to 128	8 to 32	32	1 to 64
LES400	64 to >2,048	>2,048	>256	32 to 128	64 to 2,048	64 to 2,048	8 to >128
LES431	512	128 to 256	16	2	2	4	2 to 8

a Shown is the MBEC value range for *n* = 2 biological replicates (three technical replicates each). PIP, piperacillin; ATM, aztreonam; IPM, imipenem; TOB, tobramycin; PMB, polymyxin B; CST, colistin; CIP, ciprofloxacin.

### Trends in biofilm biomass changes upon exposure to antibiotics.

**FIG 6:**
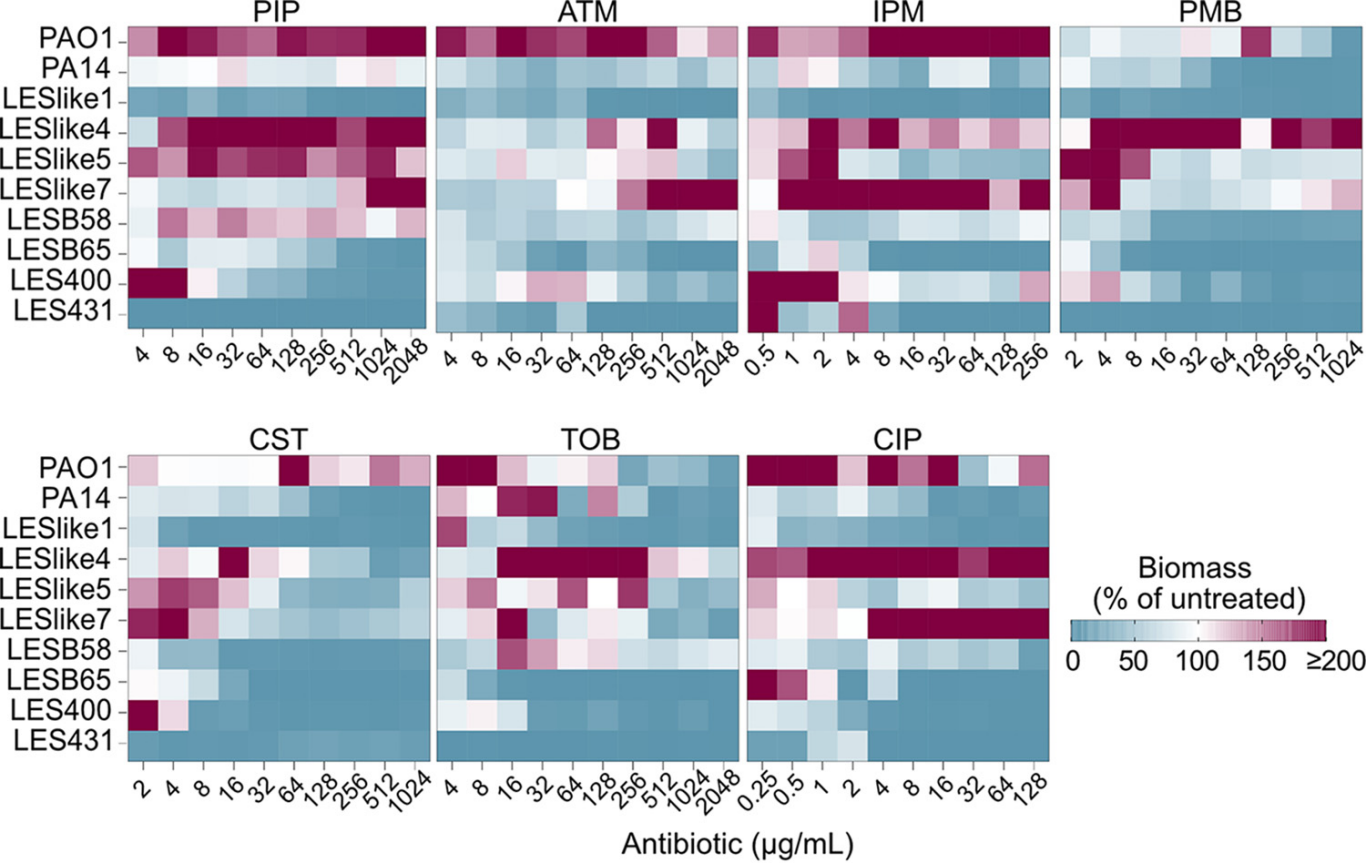
Effects of antibiotics on biofilm biomass. Heat plots show the biofilm biomass (percentage of untreated control) across the concentration ranges tested for each antibiotic in MBEC assays. Purple indicates biofilm biomass increased in the presence of antibiotic, while blue indicates that biofilm biomass was lower in the presence of antibiotic.

After the recovery phase of the MBEC assay, the biofilm biomass on the pegs can be quantified by staining with crystal violet. The biomass is then normalized to the biomass in the untreated control to determine the percentage of biomass at each antibiotic concentration. We used heat plots to visualize the percentage of biomass over the concentration ranges tested and to determine trends in biofilm biomass (Fig. 6). PAO1 showed increases in biomass compared to the untreated control at multiple consecutive concentrations near or above the MIC for all antibiotics except polymyxin B. LESlike4, LESlike5, and LESlike7 also consistently showed increases in biomass at multiple concentrations around their MICs for all antibiotics tested. LES400 showed a peak in biomass just below its MIC for several antibiotics (PIP, ATM, PMB, and CST) and showed an increase in biomass at concentrations at and above its MIC for imipenem. However, these increases in biomass did not always mean that these strains/isolates had the highest MBEC values for a given antibiotic. LESlike1 had almost no increases in biomass within the concentration ranges tested in the MBEC assays, but it still had high MBEC values (at or beyond the highest concentration tested for most antibiotics).

### Comparison of the predicted resistomes of PAO1 and LES isolates.

To identify differences in the predicted resistomes of each strain/isolate that could possibly account for variation in susceptibility values, we searched the genomes of each strain/isolate in the Comprehensive Antibiotic Resistance Database (CARD). Across all nine searches, there were 60 genes identified that are predicted to contribute to antimicrobial resistance. Of these genes, 53 were identified in all LES isolates and PAO1. A summary of the differences in the resistomes of PAO1 and the LES isolates is provided in Table 4.

**TABLE 4:** Differences in the predicted resistomes of PAO1 and LES isolates

Antimicrobial resistance gene	Description of resistome differences
*adeF*	Present in LES isolates, absent in PAO1
*basR*	Present in LES isolates, absent in PAO1
*mexF*	Present in PAO1, absent in LES isolates
*mexB*	Absent in LESlike1, LESlike4, LESlike5, and LES431
*cprS*	Absent in LESlike7 and LES400
*gyrA*	LESlike7, LESB58, and LES431 have GyrA variants that confer resistance to fluoroquinolones (residue changes D87N in LESlike7 and T83I in LESB58 and LES431).
*ampC*	CARD identified the following variants in each strain/isolate: PAO1, PDC-1; LES400, PDC-431; all other LES isolates, PDC-3. However, an alignment of AmpC sequences from the Pseudomonas Genome Database showed that the LES400 AmpC sequence is the same as those of the other LES isolates. Compared to PAO1, the LES isolates have the residue change T105A.

## DISCUSSION

Epidemic strains of P. aeruginosa that can be transferred between pwCF are a clinical concern and often display phenotypic differences from nonepidemic clinical isolates and laboratory strains. Eight LES isolates have had their genomes sequenced and fully annotated (26), and we have characterized the growth and antibiotic susceptibility profiles of these isolates. The growth curves, biofilm assays, MIC assays, and MBEC assays completed here provide basic information about these isolates and indicate the need for extensive phenotypic analysis, including phenotypic microarrays. Results for the LES isolates were compared with those from the laboratory strains PAO1 and PA14. In the planktonic growth curves, we observed lower growth rates in the LES isolates compared with PAO1, likely due to their nature to produce SCVs. Low growth rates for these eight isolates during phenotypic assays on agar plates have previously been reported (26). Slow growth has been observed in other clinical P. aeruginosa isolates, may be an adaptation as SCVs to the CF lung environment, and may contribute to antibiotic resistance (15, 31–33).

Preliminary biofilm assay results for the eight LES isolates were reported with their initial genotypic and phenotypic characterization (26). Here, we expanded on this initial characterization by measuring biofilm formation in two media at three time points to uncover potential temporal differences in biofilm formation. We also normalized biofilm formation to planktonic growth and reported quantitative values for three biological replicates with eight technical replicates each. Overall, the LES isolates tended to have similar amounts of biofilm biomass compared to PAO1. In the previously reported biofilm assays completed in lysogeny broth (LB), the LES isolates also had amounts of biofilm biomass similar to or smaller than those of PAO1 (26). Our biofilm assays and previous assays have all been completed in rich laboratory media. Additional experiments may be conducted to explore differences among the LES isolates and compared to PAO1 when biofilms are cultured under conditions mimicking those of the CF lung. Assays may be conducted in artificial sputum medium (34, 35) to see how these nutritional cues affect biofilm formation in the LES.

Our MIC assays showed that overall, the LES isolates had higher levels of resistance than PAO1 when challenged with a range of antibiotics with different targets. Some previous studies have reported MIC values for various LES isolates, including some of the isolates studied here (21–25). Various methods for determining MICs have been used in these studies, including Etest strips, the disc diffusion method, agar dilution, and the microdilution method we used. Dettman et al. (24) report the median MICs for a group of LESlike isolates for several antibiotics, including two tested here. They found that LESlike isolates had median MICs of 128 μg/mL for gentamicin and 4 μg/mL for ciprofloxacin, which are consistent with the range of MICs we observed among the LESlike isolates we tested (64 to 256 μg/mL and 1 to 8 μg/mL for gentamicin and ciprofloxacin, respectively). Salunkhe et al. (21) reported MIC values for LES400 and LES431 challenged with 12 different antibiotics using the Etest strip method. For the six antibiotics we also tested, they found lower MIC values for both isolates using the Etest method. However, some of our values are within two 2-fold dilutions of their results (three antibiotics for LES400 and four antibiotics for LES431).

To determine if there are any genomic differences among the LES isolates and PAO1 that may contribute to the patterns we observed in the MIC assays, we completed an analysis with the Comprehensive Antibiotic Resistance Database (CARD) (36). The CARD analysis was used to predict the resistome of each strain/isolate through its genome using a curated database of genes/proteins that contribute to antibiotic resistance. The CARD analysis identified some genes present/absent in all the LES isolates compared to PAO1 as well as differences among the LES isolates. However, none of these differences could be easily connected to the MIC results. For example, LESlike7, LESB58, and LES431 have a variant of GyrA that confers resistance to fluoroquinolones; however, these isolates had MIC values for ciprofloxacin similar to those of the other LES isolates. The CARD analysis also identified variants of AmpC in the LES isolates. LES400 was predicted to have a different variant of AmpC than the other LES isolates; however, a comparison of the sequences available in the Pseudomonas Genome Database indicates that it has the same protein sequence as the other LES isolates. It has been noted that LES400 has a mutation in the start codon of AmpC, and therefore the protein may not be translated (21); however, LES400 had MIC values for the tested β-lactams comparable to those of the other isolates tested. The CARD analysis did not identify any differences that could clearly explain the subset of LES isolates that had increased resistance to some of the β-lactams tested. This shows the difficulty of predicting resistance phenotypes from genomic data alone and that high levels of resistance in P. aeruginosa isolates are likely a result of multiple factors.

In the MBEC assays, we observed that resistance was increased in the biofilm mode of growth, which was expected given the role biofilm formation plays in inhibiting or slowing the action of antibiotics. The MBEC assays did reveal some differences in biofilm biomass over the concentration ranges tested. PAO1, LESlike4, LESlike5, LESlike7, and LES400 often showed increased biofilm formation in the presence of antibiotic. These increases in biomass were not specific to one type of antibiotic and were observed in multiple treatments. We have previously reported increases in biofilm biomass in MBEC assays for PAO1 (37, 38), and sub-MICs of antibiotics from multiple classes have been shown to increase biofilm formation in various Gram-negative and Gram-positive species (39). We hypothesize that the increase in biomass observed in the MBEC assays is due to increased production of EPSs. To further understand which biofilm components are responsible for these increases in biomass, fluorescence microscopy could be used to quantify cells and EPS components. In the MBEC assays, we also saw some isolates that had very little biomass, even in the untreated controls. For some antibiotics, these isolates (mainly LESB65 and LES431) had lower MBEC values than the other isolates tested. Given that these isolates did not consistently form biofilm under the conditions of the MBEC assay, even when untreated, we cannot conclude that resistance is not increased in the biofilm mode of growth for these isolates. It is possible that under different conditions, these isolates may form biofilms that are more resistant to antibiotic treatment. This highlights a limitation of the MBEC assay to compare multiple strains. While the CFU/peg values in the growth phase of the MBEC assay were similar among the tested isolates, the biofilm biomasses may not have been equivalent. Matrix components that contribute to biomass measurements play an important role in resistance to antibiotics; therefore, it may be necessary to take both factors into account when normalizing the growth phase of MBEC assays.

Overall, this work provides comprehensive susceptibility data for a number of antibiotics for a group of the best-characterized LES isolates. Our results show that isolates of the LES have high intrinsic antimicrobial resistance to a range of antibiotics when they are grown planktonically and that resistance is often increased by the biofilm mode of growth.

## MATERIALS AND METHODS

### Pseudomonas aeruginosa strains and isolates.

Laboratory strains and clinical isolates of Pseudomonas aeruginosa were used. The commonly studied laboratory strains P. aeruginosa PAO1 (40) and PA14 (41, 42) were used as comparator strains. Eight isolates of the LES were used. Four LES isolates originated from the United Kingdom, three of which were isolated from chronic CF infections: LESB58 (1996), LESB65 (2003), and LES400 (1998). The fourth U.K. LES isolate, LES431, was isolated from the non-CF parent of an individual with CF (2000). The other four LES isolates included in this study originated from individuals with CF in Ontario, Canada: LESlike1 (patient 01-022-1, Ottawa, 2005), LESlike4 (patient 03-019-10, Toronto, 2005), LESlike5 (patient 03-054-2, Toronto, 2007), and LESlike7 (patient 05-009-2, Hamilton, 2006). Stocks of each strain/isolate were made in glycerol (final concentration, 20% [vol/vol]) and stored at −80°C.

### Media and antibiotics.

Strains and isolates were streaked for isolated colonies on tryptic soy agar (TSA) (BD Difco) incubated at 37°C for 16 to 24 h. Overnight cultures were grown in Trypticase soy broth (TSB) or cation-adjusted Mueller-Hinton broth (CAMHB) (BD Difco). Antibiotics from eight classes were used in susceptibility testing against planktonic and biofilm-producing cultures (Table 1). All antibiotics were purchased from Sigma-Aldrich, except for imipenem (Gold Biotechnology, Inc.) and carbenicillin (Fisher Scientific).

### Planktonic growth curves.

Overnight cultures were diluted to an optical density at 600 nm (OD_600_) of 0.025, and 200 μL of the dilution was added to eight wells of a 96-well plate for each sample. Overnight cultures were grown in TSB or CAMHB and diluted in the same medium. Growth curves were carried out in a BioTek Synergy H1 plate reader. Plates were incubated at 37°C (gradient set to 1 to control condensation) and shaken on the slow setting of double orbital with continuous shaking. The OD_600_ was measured every 15 min for 24 h. Three biological replicates, each with eight technical replicates, were completed for growth curves in TSB and CAMHB.

### Biofilm assays.

Biofilm assays were performed as described by O’Toole (43). Biofilm assays were completed in both TSB and CAMHB and were inoculated from overnight cultures grown in the same medium. TSB or CAMHB (200 μL) was added to all wells of a 96-well plate. Overnight cultures were standardized to an OD_600_ of 1.0, and then 5 μL of the inoculum was added to eight wells for each strain/isolate. Each plate also included eight uninoculated blank wells. Plates were incubated statically at 37°C for 24, 48, or 96 h. After incubation, 120 μL of planktonic growth was removed from each well and added to a new 96-well plate. The OD_600_ of planktonic growth was measured in a Bio-Rad xMark plate reader. The wells of the biofilm assay were washed three times with phosphate-buffered saline (PBS) and then air-dried. Biofilms were stained with 0.2% (wt/vol) crystal violet (CV) (200 μL per well) for 15 min. Excess CV was removed, and the wells were washed three times in a water bath and then air-dried. The CV was solubilized with 30% acetic acid, and the absorbance was read at 590 nm (*A*_590_). Biofilm formation was normalized to the planktonic growth for each well (*A*_590_/OD_600_). One-way analysis of variance (ANOVA) with Dunnett’s multiple-comparison test (*P* < 0.05) was used to compare each strain/isolate with PAO1 at the same time point and in the same medium.

### MIC assays.

MIC assays were performed following the Clinical and Laboratory Standards Institute microdilution guidelines (29). MICs were determined in CAMHB with bacteria (2 × 10^5^ to 8 × 10^5^ CFU/mL) exposed to antibiotics for 20 h at 37°C, after which the OD_600_ was measured (Bio-Rad xMark plate reader). Three biological replicates, with three technical replicates each, were completed. The MIC was defined as the lowest concentration of antibiotic where no visual growth was observed.

### MBEC assays.

The susceptibility of LES biofilms was determined using a minimum biofilm eradication (MBEC) assay (44). MBEC assays are divided into a growth phase, challenge phase, and recovery phase. Biofilms are first grown on the pegs of a specialized lid for 96-well plates (growth phase) before being exposed to antibiotics (challenge phase). After challenge, the biofilms are allowed to recover in fresh medium, where they can establish a planktonic population (recovery phase). Planktonic growth in the wells of the recovery phase indicates biofilm viability and is used to determine the MBEC value. MBEC assays were performed as described by Habash et al. (37, 38), with minor modifications. MBEC assays were completed in CAMHB, and MBEC plates (Innovotech) were incubated at 37°C with shaking at 100 rpm for all phases of the assay. Overnight cultures were standardized to an OD_600_ of 1.0, and 5 μL was added to the appropriate wells containing 145 μL of CAMHB. Plates were then incubated for 24 h in the growth phase. To compare the different strains/isolates, we first established that they each formed biofilms with similar CFU/peg values in the growth phase of the assay as done in previous studies working with multiple strains (30). To measure the CFU/peg after the growth phase, the pegs were washed in sterile PBS and moved to a fresh 96-well plate containing 200 μL sterile PBS, and the plate was sonicated for 30 min in a water bath to remove biofilm cells from the pegs. Samples from each well were then serially diluted in PBS, 10 μL from each dilution was spotted onto TSA plates, which were incubated at 37°C overnight, and then colonies were counted.

After the growth phase in a complete MBEC assay, the pegs were washed in sterile PBS and transferred to a challenge plate containing 2-fold dilutions of antibiotics. After 20 h, the pegs were washed in sterile PBS and transferred to a recovery plate containing fresh CAMHB. After 24 h, the OD_600_ of the recovery plates was measured to determine biofilm viability after antibiotic challenge (37). The MBEC was defined as the lowest concentration of antibiotic that resulted in no visible growth. Following recovery, the biomass on the pegs was determined by staining with CV as in the biofilm assays. Pegs were washed in PBS, air-dried, stained with 0.2% CV for 15 min, washed in a water bath, and air-dried, and then the CV was solubilized in 30% acetic acid.

### CARD resistome prediction.

The resistomes of PAO1 and LES isolates were predicted using the Resistance Gene Identifier (RGI) (v5.2.0) of the Comprehensive Antimicrobial Resistance Database (CARD) (v3.1.4) (36). The GenBank accession numbers for the genomes of each strain/isolate were searched in the RGI using the default settings (perfect and strict hits only and with loose hits with ≥95% identity not nudged to strict hits). The following accession numbers were searched: PAO1, NC_002516.2; LESlike1, CP006984.1; LESlike4, CP006985; LESlike5, CP006980.1; LESlike7, CP006981.1; LESB58, NC_011770.1; LESB65, CP006983.1; LES400, CP006982.1; LES431, CP006937.1. We then compared the results of each search to identify differences in the resistomes between PAO1 and the LES isolates.
