# Supplementary material for: Liverpool Epidemic Strain Isolates of Pseudomonas aeruginosa Display High Levels of Antimicrobial Resistance during Both Planktonic and Biofilm Growth
Source: Microbiol Spectr. 2022 Jun 6;10(3):e01024-22. doi: 10.1128/spectrum.01024-22 (PMC9241699; doi:10.1128/spectrum.01024-22)
Supplement: Supplemental file 1 — Supplemental material. Download spectrum.01024-22-s0001.pdf, PDF file, 0.2 MB [file spectrum.01024-22-s0001.pdf]

SUPPLEMENTAL FIGURE

Liverpool Epidemic Strain Isolates of *Pseudomonas aeruginosa* display high levels of antimicrobial resistance during both planktonic and biofilm growth

Mara C. Goodyear, Nicole E. Garnier, Roger C. Levesque and Cezar M. Khursigara

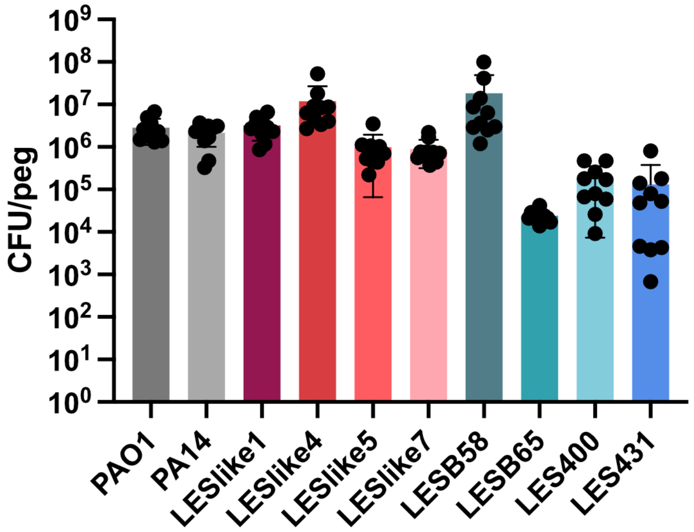

Figure S1 MBEC assay biofilm growth comparison. Biofilm growth after 24 h at 37 °C and 100 rpm in CAMHB was removed from pegs by sonication and the CFU/peg was determined by serial dilution and plating. CFU/peg values are reported as the mean ± SD for ten pegs (points indicate values for each peg, five pegs each from two MBEC assays).
